# Current advances in cancer vaccines targeting NY-ESO-1 for solid cancer treatment

**DOI:** 10.3389/fimmu.2023.1255799

**Published:** 2023-09-05

**Authors:** Hong Zhou, Yipeng Ma, Fenglan Liu, Bin Li, Dongjuan Qiao, Peigen Ren, Mingjun Wang

**Affiliations:** ^1^ Center for Energy Metabolism and Reproduction, Shenzhen Institutes of Advanced Technology, Chinese Academy of Sciences, Shenzhen, China; ^2^ Department of Research and Development, Shenzhen Innovation Immunotechnology Co., Ltd, Shenzhen, China; ^3^ Department of Research and Development, Shenzhen Institute for Innovation and Translational Medicine, Shenzhen, China

**Keywords:** CTA, NY-ESO-1, cancer vaccine, immunotherapy, solid cancer

## Abstract

New York-esophageal cancer 1 (NY-ESO-1) belongs to the cancer testis antigen (CTA) family, and has been identified as one of the most immunogenic tumor-associated antigens (TAAs) among the family members. Given its ability to trigger spontaneous humoral and cellular immune response and restricted expression, NY-ESO-1 has emerged as one of the most promising targets for cancer immunotherapy. Cancer vaccines, an important element of cancer immunotherapy, function by presenting an exogenous source of TAA proteins, peptides, and antigenic epitopes to CD4^+^ T cells *via* major histocompatibility complex class II (MHC-II) and to CD8^+^ T cells *via* major histocompatibility complex class I (MHC-I). These mechanisms further enhance the immune response against TAAs mediated by cytotoxic T lymphocytes (CTLs) and helper T cells. NY-ESO-1-based cancer vaccines have a history of nearly two decades, starting from the first clinical trial conducted in 2003. The current cancer vaccines targeting NY-ESO-1 have various types, including Dendritic cells (DC)-based vaccines, peptide vaccines, protein vaccines, viral vaccines, bacterial vaccines, therapeutic whole-tumor cell vaccines, DNA vaccines and mRNA vaccines, which exhibit their respective benefits and obstacles in the development and application. Here, we summarized the current advances in cancer vaccines targeting NY-ESO-1 for solid cancer treatment, aiming to provide perspectives for future research.

## 1 Introduction

The NY-ESO-1 gene is located in chromosome Xq28 with a total length of 747 bp and encodes an 18-KDa protein (1). NY-ESO-1 is a 180-amino acid protein with a glycine-rich N-terminal region and a strongly hydrophobic C-terminal region that contains a conserved Pcc-1 domain (2). The expression of this gene is predominantly restricted to a variety of solid tumors, germ cells and placental cells, exhibiting little or no expression in normal adult somatic tissues (3). The positive expression rate of NY-ESO-1 varies among different solid tumors. For instance, myxoid and round cell liposarcoma shows the highest expression rate (89-100%) of NY-ESO-1 by immunohistochemistry with the monoclonal antibodies ES121 and E978 (4), followed by neuroblastoma (82%), synovial sarcoma (80%), melanoma (46%), and epithelial ovarian cancer (43%) (5–9). The highly restricted expression of NY-ESO-1 antigen in normal tissues (male germ cells) and its widespread expression in different tumor types make it a promising candidate target for tumor immunotherapy. However, the heterogeneous expression pattern of NY-ESO-1 in tumor tissues may affect the treatment effect of immunotherapy. The most homogeneous expression of NY-ESO-1 has been reported in synovial sarcomas (70%) (6), making these tumors a promising candidate for immunotherapy targeting the NY-ESO-1 antigen. Multiple clinical trials are currently in progress to explore the potential of NY-ESO-1 as a target for cancer immunotherapy. One strategy involves the use of genetically modified T cells that are engineered to specifically recognize and eliminate cancer cells expressing NY-ESO-1. Another strategy is to use cancer vaccines or other treatments to induce and activate the endogenous immune system to recognize and eliminate NY-ESO-1-positive cancer cells (3).

Cancer vaccines are a type of vaccine that is designed to activate the immune system to recognize and attack cancer cells (10). Unlike traditional vaccines, which are designed to prevent infectious diseases, cancer vaccines are used to treat cancer or to prevent cancer from recurring after treatment (10). Although cancer vaccines have shown promising results in preclinical and clinical trials, they are not yet widely available or approved for use as a standard cancer treatment (11, 12). Sipuleucel-T (Provenge, or APC8015) is currently only one FDA-approved therapeutic cancer vaccine, which is a DC-based vaccine to treat prostate cancer that has metastasized (11, 13). Cancer cells may evade immune attack induced by cancer vaccines through various mechanisms, including antigen depletion, alterations in antigen processing and the decreased surface expression of human leukocyte antigen class I (HLA-I) molecules (11). In addition, since the successful treatment with cancer vaccines depends on the activation of T cells, an effective response may not occur if the patient’s cancer cells have an insufficient capacity for tumor antigen processing and presentation (14, 15). Therefore, future studies may focus on combining appropriate cancer vaccines with innovative immunomodulatory strategies and standard-of-care treatment for overcoming impaired antitumor responses and therapy resistance (11). Research in this area is ongoing, and it is hoped that cancer vaccines will one day become an important tool in fighting against cancer. Here, we summarized the current advances in cancer vaccines targeting NY-ESO-1 for solid cancer treatment, aiming to provide perspectives for future research.

## 2 Cancer vaccines targeting NY-ESO-1

**Figure 1:**
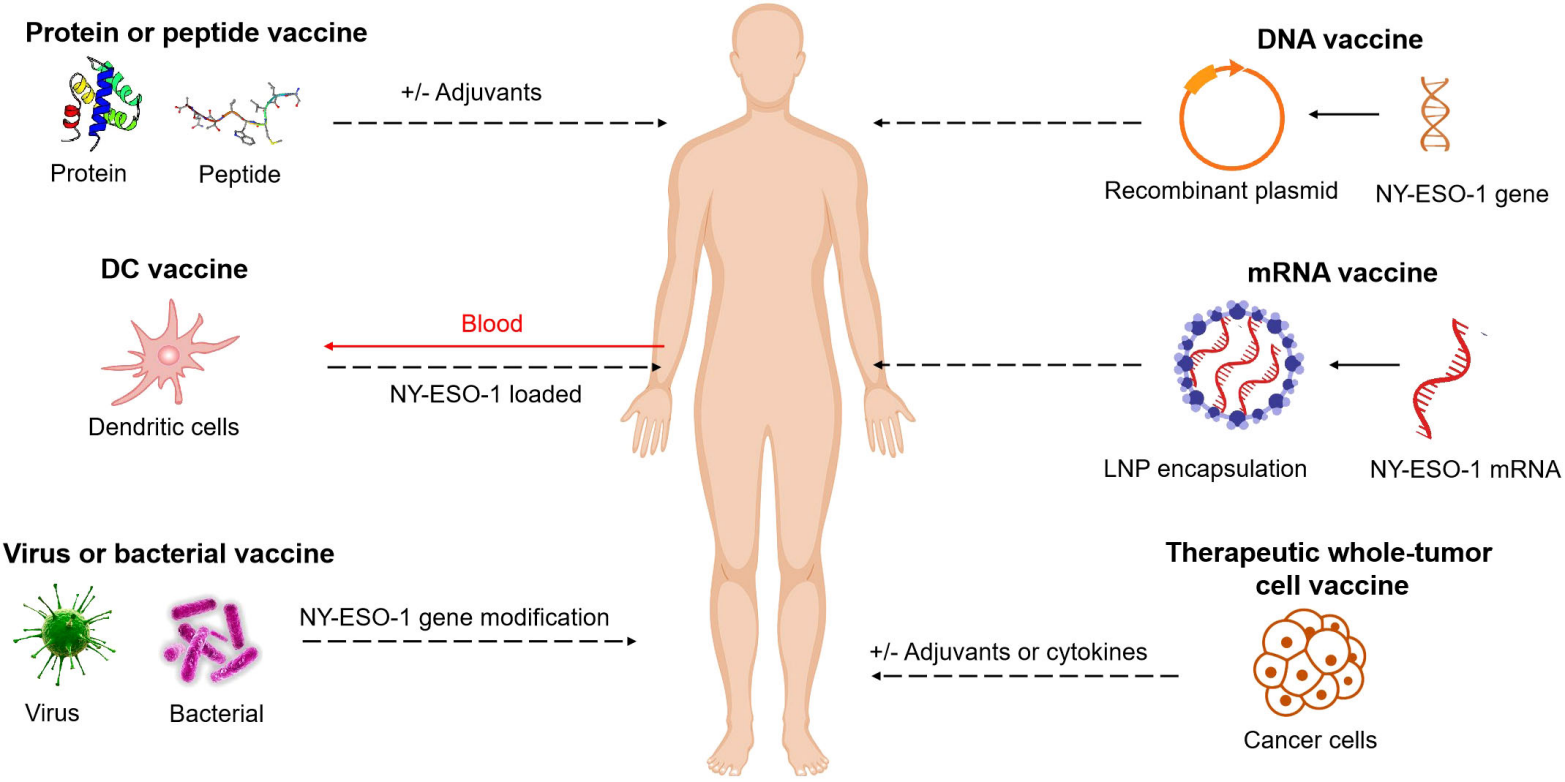
NY-ESO-1-based cancer vaccine approaches. Vaccines include short peptides, full length proteins (with and without adjuvants), DC vaccines, viruses or bacterial vaccines with NY-ESO-1 gene modification, DNA or mRNA vaccines (with NY-ESO-1 in sequences), therapeutic whole-tumor cell vaccines. These elements can be modified, added to adjuvants, or combined together.

The efficacy of cancer vaccines targeting NY-ESO-1 antigen has been extensively investigated using various formulations, including DC vaccines, peptide vaccines, protein vaccines, viral vaccines, bacterial vaccines, therapeutic whole-tumor cell vaccines, DNA vaccines and mRNA vaccines. These studies have validated the safety of the NY-ESO-1 antigen-targeted vaccines and demonstrated their immunogenicity. (**Figure 1**)

### 2.1 Bacterial vaccines

Bacteria possess pathogen-associated molecular patterns (PAMPs) which can be recognized by pattern recognition receptors (PRRs) like Toll-like receptors (TLRs)on immune cells and trigger an innate immune response against the bacteria. This innate immune response can also influence the adaptive immune system, making attenuated or avirulent recombinant bacterial vectors highly effective in stimulating targeted and durable immune responses to the antigens carried in the bacterial vectors (16, 17). These bacterial vectors not only produce and deliver antigens, but also act as immune-stimulating adjuvants. Several preclinical and clinical trials had investigated the effectiveness of bacterial vaccines targeting NY-ESO-1. The *Salmonella typhimurium* vaccine was engineered to deliver NY-ESO-1 antigen through a type III protein secretion system, and was proved to be effective in mice and could elicit NY-ESO-1-specific CD8^+^ and CD4^+^ T cells responses *in vitro* (18). Interestingly, the antigen-specific T cell responses induced by *S. typhimurium*-NY-ESO-1 vaccine were resistant to suppression by CD4^+^CD25^+^ Treg cells (19). In addition, a phase I clinical trial (NCT01967758) on a live-attenuated listeria monocytogenes vaccine (ADU-623), which was engineered to express NY-ESO-1 and EGFRvIII for the treatment of glioma, was performed, but clinical results for safety and efficacy have not been reported yet.

In contrast to viral vaccine vectors, bacterial vectors under development for human use are susceptible to antibiotics, enabling treatment in case of adverse reactions during extensive vaccination trials (20). This characteristic has led to increased interest in bacterial vectors for the development of cancer vaccines.

### 2.2 DC vaccines

DCs are the dominant antigen-presenting cells in the immune system and play a crucial role in initiating adaptive immune responses (21). Extensive clinical trials have been conducted, particularly in patients with advanced melanoma, to investigate the potential of DCs in immunotherapy (22–24). In these trials, patients were treated with autologous DCs, which were loaded with antigens in order to induce specific T cell responses against cancer cells. DCs can be loaded with the antigens in various forms, including peptides, whole protein, tumor lysate, apoptotic debris or complexed with antibody (25). Moreover, antigens encoded by mRNA or cDNA is particularly appealing as it offers a more straightforward approach compared to the use of externally provided peptides and proteins. This approach simplifies the isolation and utilization of antigens in clinical settings (25–27). However, the transfection of DCs with cDNA encoding antigens has not been demonstrated effectiveness (28). In contrast, the transfection of DCs with mRNA encoding antigens has been found to be a highly efficient method for antigen loading (26, 28).

Several ongoing clinical trials are exploring the safety and efficacy of NY-ESO-1 peptide-pulsed DCs, either alone or in combination with NY-ESO-1 protein vaccine and TLR agonists. The information of these trials are included in **Table 1**. As an illustrative example, in a phase IIa trial (NCT02692976), DCs were activated with protamine/mRNA and loaded with TAAs including NY-ESO-1, MAGE-C2 and MUC-1. After vaccination, antigen-specific T cells were detectable in peripheral blood of 12 out of 21 patients (57%). Specifically, NY-ESO-1, MAGE-C2 and MUC-1 specific T cells were identified in the blood of 10 out of 21 patients (48%), 4 out of 21 patients (19%), and 2 out of 21 patients (10%), respectively. Importantly, all patients tolerated the DC vaccines well, with only grade 1-2 toxicity reported (29). Additional trials have been performed to evaluate the safety and feasibility of combining NY-ESO-1 peptide-pulsed DC vaccine with NY-ESO-1 transduced T cell therapy and Nivolumab in advanced solid cancers (NCT02775292, **Table 1**). This combination therapy demonstrated temporary anti-tumor activity. Reconstitution of NY-ESO-1-specific T cells in the peripheral blood reached its peak within two weeks, indicating rapid *in vivo* expansion. The transferred lymphocytes exhibited predominantly an effector memory phenotype, without progressing to exhaustion or terminal differentiation. On-treatment biopsy revealed complete loss of NY-ESO-1 expression, accompanied by extensive methylation of the promoter sequence (30). The combination of NY-ESO-1 pulsed DC vaccination named CMB305 with Atezolizumab has been investigated in patients with sarcoma (NCT02609984, **Table 1**). While the combination of CMB305 and atezolizumab did not show significant improvements in progression-free survival (PFS) or overall survival (OS) compared to atezolizumab alone, some patients exhibited indications of an anti-NY-ESO-1 immune response and showed better outcomes in imaging assessments (31).

**Table 1:** Clinical trials of cancer vaccines based on NY-ESO-1.

Type of vaccines	Diseases	Trials	NCT number	Status	Locations	Start year	Characteristics
Bacterial vaccine	Astrocytic tumors, glioblastoma multiforme, anaplastic astrocytoma, brain tumor	Phase 1	NCT01967758	Completed	U.S.	2013	ADU-623, a Live-attenuated Listeria Monocytogenes Strain (ΔactA/ΔinlB) Expressing the EGFRvIII-NY-ESO-1 Vaccine
DC vaccine	Sarcoma|melanoma|non-small cell lung cancer|ovarian cancer	Phase 1b	NCT02387125	Terminated (This study did not meet the efficacy objective)	U.S.	2015	CMB305 (peptide-pulsed DC vaccine LV305 + G305 recombinant NY-ESO-1 protein vaccine)| TLR4 agonist (G100)
DC vaccine	Ovarian Cancer	Phase 1	NCT04739527	Recruiting	Netherlands	2021	allogeneic dendritic cell vaccine (DCP-001)
DC vaccine	Non Small Cell Lung Cancer	Phase 1/2	NCT03970746	Recruiting	Belgium,France,Germany,Netherlands,Poland	2019	PDC*lung01, Associated or Not With Anti-PD-1
DC vaccine	Sarcoma	Phase 2	NCT02609984	Terminated (This study did not meet the efficacy objective)	U.S.	2015	CMB305 (peptide-pulsed DC vaccine LV305 + G305 recombinant NY-ESO-1 protein vaccine)| with Atezolizumab to Atezolizumab Alone
DC vaccine	Acute Myelogenous Leukemia	Phase 1	NCT01483274	Withdrawn (Adult patient population barriers.)	U.S.	2011	mature DC will be pulsed with overlapping peptides mixes derived from full-length NY-ESO-1, MAGE-A1, and MAGE-A3
DC vaccine	Neuroblastoma,Rhabdomyosarcoma,Osteogenic Sarcoma	Phase 1	NCT00944580	Withdrawn (unexpectedly low screening results leading to poor accrual)	–	2009	MAGE-A1, MAGE-A3, and NY-ESO-1 Vaccine.A regimen of three vaccines every two weeks. Each vaccine will contain 3,000,000-5,000,000 peptide pulsed dendritic cells.
DC vaccine	Solid Tumors expressing NY-ESO-1	Phase 1	NCT02775292	Completed	U.S.	2016	NY-ESO-1(157-165) Peptide-pulsed Autologous Dendritic Cell Vaccine, nivolumab, NY-ESO-1 reactive TCR retroviral transduced autologous PBL
DC vaccine	Prostate cancer	Phase 2a	NCT02692976	Completed	Netherlands	2016	Multi peptide (NY-ESO-1, MAGE-C2 and MUC1) -pulsed myeloid and plasmacytoid DC vaccine
DC vaccine	Melanoma	Phase 2	NCT02334735	Completed	U.S.	2015	Multi peptide (NY-ESO-1 and Melan-A/MART-1) - pulsed DC vaccine, Poly-ICLC ;;DCs as an adjuvant for NY-ESO-1 and Melan-A/MART-1 peptides compared to Montanide^®^
DC vaccine	Gliomas, Medulloblastoma, Neuroectodermal Tumors, Primitive	Phase 1/2	NCT02332889	Terminated (Transition to a different immunotherapy strategy in the future at our institution)	U.S.	2015	Vaccine (autologous dendritic cells) Peptide pulsed DC
DC vaccine	Progressive Solid Malignancies, Refractory Solid Malignancies, Cancer	Phase 1/2	NCT02224599	Terminated(Sponsor decision to terminate study due to poor accrual)	U.S.	2014	TAPA-pulsed DC vaccine,Cyclophosphamide
DC vaccine	Malignancies	Phase 1	NCT02070406	Terminated (low accrual)	U.S.	2014	NY-ESO-1157-165 peptide pulsed DC vaccine, TCR-T, Ipilimumab
DC vaccine	Sarcoma, Neoplasms, Connective and Soft Tissue	Phase 1/2	NCT01883518	Unknown	Russian Federation	2013	Allogeneic tumor lysate (NY-ESO-1, MAGE-A3) -pulsed DC vaccine
DC vaccine	Malignant Neoplasm	Phase 2	NCT01697527	Active, not recruiting	U.S.	2012	NY-ESO-1 (157-165) peptide pulsed dendritic cells (DC), TCR-T
DC vaccine	Neuroblastoma and Sarcoma	Phase 1	NCT01241162	Completed	U.S.	2010	For vaccine production, mature DC will be pulsed with overlapping peptides mixes derived from full-length NY-ESO-1, MAGE-A1, and MAGE-A3.
DC vaccine	Non Small Cell Lung Cancer	Phase 2	NCT01159288	Completed	France	2010	Tumor Antigen-loaded Dendritic Cell-derived Exosomes (CSET 1437) Dex2
DC vaccine	Melanoma (Skin)	Phase 1	NCT00798629	Completed	U.S.	2008	Adenovirus CCL-21 Transduced MART-1/gp100/Tyrosinase/NY-ESO-1 Peptide-Pulsed Dendritic Cells
DC vaccine	Chemotherapy-naive Metastatic Melanoma	Phase 1	NCT00313508	Completed	U.S.	2006	MART-1/gp100/Tyrosinase/NY-ESO-1 Peptide-Pulsed Dendritic Cells Matured Using Cytokines With Autologous Lymphocyte Infusion With or Without Escalating Doses of Fludarabine
DC vaccine	Melanoma, non-small cell lung cancer, sarcoma	Phase 1	NCT02122861	Completed	U.S.	2014	DC lentiviral vector vaccine (LV305)
DC vaccine	Synovial Sarcoma, Cancer, Soft Tissue Sarcoma,Sarcoma, Metastatic Sarcoma	Phase 3	NCT03520959	Terminated (Study was terminated due to sponsor’s decision)	U.S.,Canada	2018	LV305+G305,CMB305
DNA vaccine	Prostate Cancer, Bladder Cancer, Non-small Cell Lung Cancer, Esophageal Cancer, Sarcoma	Phase 1	NCT00199849	Completed	U.S.	2005	NY-ESO-1 Plasmid DNA (pPJV7611) Cancer Vaccine given by particle-mediated epidermal delivery (PMED)
mRNA vaccine	Melanoma	Phase 1	NCT02410733	Active, not recruiting	Germany	2015	tetravalent RNA-lipoplex cancer vaccine(BNT111)
mRNA vaccine	Metastatic Non-small Cell Lung Cancer, NSCLC	Phase 1/2	NCT03164772	Completed	U.S.	2017	F2410 coding for NY-ESO-1, mRNA vaccine called CV9202
mRNA vaccine	Melanoma	Phase 2	NCT04526899	Recruiting	U.S.,Australia,Germany,Italy,Poland,Spain,United Kingdom	2020	BNT111 and Cemiplimab
mRNA vaccine	Non Small Cell Lung Cancer	Phase 1/2a	NCT00923312	Completed	Switzerland, Germany	2009	CV9201, a mRNA vaccnine encoding 5 NSCLC antigens (NY-ESO-1, MAGE-C1/2, survivin, trophoblast glycoprotein (5T4)
Protein vaccine	Neoplasms	Phase 1	NCT00106158	Completed	Japan	2005	Complex of NY-ESO-1 Protein and Cholesterol-bearing Hydrophobized Pullulan (CHP)
Protein vaccine	Esophageal Cancer	Phase 1	NCT01003808	Completed	Japan	2009	injected as a complex with cholesteryl pullulan (CHP), forming nano-particles (IMF-001)
Protein vaccine	Esophageal Cancer, Lung Cancer, Stomach Cancer,Breast Cancer,Ovarian Cancer	Phase 1	NCT00291473	Completed	Japan	2006	CHP-HER2 and CHP-NY-ESO-1 Protein With OK-432
Protein vaccine	Fallopian tube carcinoma, ovarian carcinoma, primary peritoneal carcinoma	Phase 1/2b	NCT02166905	Completed	U.S.	2014	DEC-205/NY-ESO-1 Fusion Protein (CDX-1401),IDO1 inhibitor (Epacadostat)
Protein vaccine	Solid Tumors	Phase 1	NCT01522820	Completed	U.S.	2012	DEC-205/NY-ESO-1 Fusion Protein CDX-1401 With or Without Sirolimus
Protein vaccine	Melanoma	Phase 2	NCT02129075	Completed	U.S.	2014	DEC-205/NY-ESO-1 Fusion Protein vaccine (CDX-1401)|Recombinant Flt3 Ligand (CDX-301)
Protein vaccine	Advanced Malignancies	Phase 1/2	NCT00948961	Completed	U.S.	2009	CDX-1401 in combination with Resiquimod and/or Poly-ICLC
Protein vaccine	Advanced Solid Tumor	Phase 1/2	NCT02661100	Withdrawn (Drug unavailable)	–	2016	CDX-1401 in Combination With Poly-ICLC and Pembrolizumab
Protein vaccine	Recurrent Ovarian, Fallopian Tube, or Primary Peritoneal Cancer	Phase 1/2	NCT03206047	Active, not recruiting	U.S.	2017	Atezolizumab, Guadecitabine, and CDX-1401 Vaccine
Protein vaccine	Myelodysplastic Syndrome or Acute Myeloid Leukemia	Phase 1	NCT03358719	Completed	U.S.	2017	DEC-205/NY-ESO-1 Fusion Protein CDX-1401, Poly ICLC, Decitabine, and Nivolumab
Protein vaccine	Solid Tumor	Phase 1	NCT01234012	Completed	U.S.	2010	IMF-001
Protein vaccine	Tumors that often express NY-ESO-1	Phase 1	NCT00299728	Completed	U.S.	2006	NY-ESO-1 recombinant protein mixed with immune adjuvants CpG 7909 and Montanide ISA-51 VG
Protein vaccine	Melanoma	Phase 2	NCT00199901	Completed	Australia, New Zealand, United Kingdom	2005	NY-ESO-1 ISCOMATRIX^®^ Vaccine and ISCOMATRIX^®^ Adjuvant
Protein vaccine	Prostate Cancer	Phase 1	NCT00292045	Completed	Germany,Switzerland	2006	NY-ESO-1 Protein Combined With CpG 7909 (adjuvant)
Protein vaccine	Melanoma	Phase 2	NCT00518206	Completed	Australia	2007	NY-ESO-1 protein+ISCOM adjuvant vaccine/NY-ESO-1 ISCOM vaccine+cyclophosphamide
Protein vaccine	Melanoma	Phase 1/2	NCT01079741	Completed	U.S.	2010	TLR3 Agonist Poly-ICLC as an Adjuvant for NY-ESO-1 Protein Vaccination With or Without Montanide ^®^ ISA-51 VG
Protein vaccine	Tumors	Phase 1	NCT00821652	Completed	U.S.	2009	Topical Resiquimod as an Adjuvant for NY-ESO-1 Protein+Montanide Vaccination
Protein vaccine	Transitional Cell Carcinoma	Phase 1	NCT00070070	Completed	U.S.	2003	TICE^®^-strain BCG+TICE^®^-strain BCG+NY-ESO-1 protein
Protein/peptide vaccine	NY-ESO-1-expressing Tumors	Phase 1	NCT00819806	Completed	U.S.	2009	peptide vaccine or protein vaccine in association with CpG 7909 and cyclophosphamide
Protein vaccine	Malignant Melanoma	Phase 1	NCT00142454	Completed	U.S.	2005	NY-ESO-1 Protein Vaccine With Imiquimod as Adjuvant
Protein/peptide vaccine	Unresectable or metastatic melanoma	Phase 1	NCT01810016	Terminated(Low recruitment due to change in standard of care)	U.S.,Australia	2013	NY-ESO-1 protein vaccine With Ipilimumab ;NY-ESO-1 OLP4 vaccine with poly-ICLC and montanide
Protein vaccine	Fallopian tube cancer, ovarian cancer, primary peritoneal cancer	Phase 1	NCT02833506	Withdrawn (production of adjuvant to be used with vaccine was discontinued by sponsor)	U.S.	2016	Recombinant NY-ESO-1 Protein vaccine| mTOR inhibitor (Sirolimus)
Peptide vaccine	Recurrent Fallopian Tube Cancer, Recurrent Ovarian Epithelial Cancer, Recurrent Primary Peritoneal Cavity Cancer	Phase 1	NCT01673217	Completed	U.S.	2012	given together with decitabine, pegylated liposomal doxorubicin hydrochloride
Peptide vaccine	Myelodysplastic syndrome, acute myeloid leukemia	Phase 1	NCT02750995	Completed	Denmark	2016	Multi peptide vaccine (NY-ESO-1, PRAME, MAGE-A3, WT-1) in Combination With Azacitidine
Peptide vaccine	Cancer neoplasm	Phase 1	NCT00199836	Completed	Germany	2005	NY-ESO-1b Peptide Plus CpG 7909 and Montanide^®^ ISA-51
Peptide vaccine	Epithelial Ovarian, Fallopian Tube, or Primary Peritoneal Cancer	Phase 1	NCT00616941	Completed	U.S.	2008	NY-ESO-1 OLP4 + Montanide + Poly-ICLC
Peptide vaccine	Ovarian Cancer, Fallopian Tube, Primary Peritoneal Cancer, Recurrent Ovarian Cancer	Phase 1	NCT02737787	Completed	U.S.	2016	Concomitant WT1 Analog Peptide Vaccine or NY-ESO-1 Overlapping Peptides Vaccine in Combination With Nivolumab
Peptide vaccine	Ovarian Cancer, Primary Peritoneal Cavity Cancer, Fallopian Tube Cancer	Phase 1	NCT00066729	Completed	U.S.	2003	NY-ESO-1b Peptide Plus Montanide ^®^ ISA-51
Peptide vaccine	Prostate Cancer	Phase 1	NCT00616291	Completed	U.S.	2008	NY-ESO-1/LAGE-1 HLA class I/II peptide vaccine
Peptide vaccine	Multiple Myeloma	Phase 2/3	NCT00090493	Completed	U.S.	2004	NY-ESO-1 peptide and GM-CSF adjuvant
Peptide vaccine	Ovarian Cancer	Phase 2	NCT05479045	Not yet recruiting	U.S.	2022	NY-ESO-1 Peptide vaccine+Nivolumab
Peptide vaccine	Melanoma	Phase 1	NCT00112242	Completed	Switzerland	2005	vaccination with melanoma antigen peptides [Melan-A/Mart-1 (both EAA and ELA), NY-ESO-1b analog, Long NY-ESO-1 LP and MAGE-A10] and Montanide, CpG adjuvants and low dose rIL-2
Peptide vaccine	Melanoma	Phase 1	NCT01008527	Completed	U.S.	2009	Poly IC : LC and NY-ESO-1/gp100 Peptides Either Emulsified With Montanide ISA 51 or in Aqueous Solution With Escalating Doses of CP 870,893
Peptide vaccine	Melanoma (Skin)	Early Phase 1	NCT00470379	Completed	U.S.	2007	Transcutaneous (Topical) Peptide Immunization With NY-ESO-1b (SLLMWITQC) Peptide Using Resiquimod as an Immune Adjuvant
Peptide vaccine	Melanoma (Skin)	Phase 2	NCT00020397	Completed	U.S.	2003	NY-ESO-1 peptide vaccine+aldesleukin
Peptide vaccine	Melanoma (Skin)	Phase 1	NCT00037037	Unknown	U.S.	2003	Vaccine Therapy With or Without Sargramostim
Peptide vaccine	resected high-risk melanoma	Phase 1/2	NCT02126579	Unknown	U.S.	2014	long peptide vaccine (LPV7) plus toll-like receptor (TLR) agonists with or without incomplete Freund’s adjuvant (IFA)
Peptide vaccine	Malignant Melanoma	Phase 1/2	NCT00145158	Terminated (Availability of Investigational agent)	Belgium	2005	8 HLA-A2-restricted peptides and Montanide ISA51 or CpG 7909
Peptide vaccine	Melanoma (Skin)	Phase 1	NCT01176461	Completed	U.S.	2010	Multiple Class I Peptides & Montanide ISA 51 VG w Escalating Doses of Anti-PD-1 ab BMS936558
Peptide vaccine	Melanoma (Skin)	Phase 1	NCT01176474	Completed	U.S.	2010	Vaccine Combining Multiple Class I Peptides and Montanide ISA 51VG With Escalating Doses of Anti-PD-1 Antibody Nivolumab or Ipilimumab With Nivolumab
Peptide vaccine	Melanoma	Phase 1/2	NCT01308294	Terminated (Low enrollment rate)	Switzerland	2011	vaccination with tumor antigenic peptides and both IMP321/LAG-3 and Montanide adjuvants
Peptide vaccine	Sarcoma	Phase 1	NCT00027911	Terminated (Departure of PI)	U.S.	2003	NY-ESO-1 peptide vaccine+sargramostim
Viral vaccine	Fallopian Tube Cancer, Ovarian Epithelial Cancer, Primary Peritoneal Cavity Cancer	Phase 1	NCT01536054	Completed	U.S.	2012	ALVAC(2)-NY-ESO-1 (M)/TRICOM vaccine| mTOR inhibitor (Sirolimus)
Viral vaccine	Fallopian Tube Cancer, Ovarian Cancer, Peritoneal Cavity Cancer	Phase 1	NCT00803569	Completed	U.S.	2008	ALVAC(2)-NY-ESO-1(M)/TRICOM (VCP2292),GM-CSF sargramostim
Viral vaccine	Epithelial Ovarian, Fallopian Tube, or Primary Peritoneal Cancer	Phase 1/2b	NCT01982487	Withdrawn	–	2013	ALVAC(2)-NY-ESO-1 (M)/TRICOM vaccine+IDO1 Inhibitor INCB024360
Viral vaccine	Fallopian Tube cancer, Ovarian Cancer, Peritoneal Cavity Cancer	Phase 2	NCT00112957	Completed	U.S.	2005	Recombinant Vaccinia-NY-ESO-1 (rV-NY-ESO-1) and Recombinant Fowlpox-NY-ESO-1 (rF-NY-ESO-1)
Viral vaccine	Non-small Cell Lung Cancer (NSCLC)	Phase 1/2a	NCT04908111	Recruiting	United Kingdom	2021	Chimpanzee Adenovirus Oxford 1 (ChAdOx1) and Modified Vaccinia Ankara (MVA) Vaccines+Chemotherapy and an Immune Checkpoint Inhibitor

Another approach to target cancer vaccines toward DCs is through TLRs. A notable example is the utilization of a NY-ESO-1 encoding LV305 lentivirus, which specifically targets DCs through TLR3 and TLR7. This approach induced a robust cellular immune response and resulted in significant disease regression in one patient with metastatic, treatment-refractory synovial sarcoma (32). The mentioned case report is part of a completed phase I clinical trial (NCT02122861, **Table 1**) investigating the intradermal administration of NY-ESO-1-specific lentiviral DC-targeting in various cancer types, including melanoma, non-small cell lung cancer (NSCLC), ovarian cancer, and sarcoma.

DC vaccines have the capability to incorporate tumor proteins, mRNAs, and DNAs, enabling the rapid activation of T cells independent of the patients’ HLA type. Moreover, DC-based vaccines have demonstrated a favorable safety profile. However, the clinical effectiveness of NY-ESO-1 peptide-pulsed DC vaccines has not consistently met expectations, which may be due to a low proportion of patients achieving specific anti-NY-ESO-1 immune responses. Additionally, the complexity of the procedure poses challenges in ensuring the reliability of DC vaccines.

### 2.3 DNA vaccines

DNA vaccines are a type of vaccine that contain a DNA fragment encoding a specific protein antigen. These vaccines work by activating the body’s immune responses through the expression of the antigen, which helps to fight against the tumor development and progression. In particular, DNA vaccines targeting NY-ESO-1 have been studied in both preclinical and clinical trials as a potential immunotherapy.

A phase I clinical trials (NCT00199849) on NY-ESO-1 DNA vaccine (pPJV7611, plasmid) has been completed for tumor vaccination (33). The vaccine was administered safely through particle-mediated epidermal delivery (PMED) and evaluated for safety and immunogenicity in patients with confirmed NY-ESO-1 antigen expression. Among 15 patients who had no antigen-specific immune responses prior to vaccination, 14 (93%) developed antigen-specific CD4^+^ T cell responses, and 5 patients (33%) developed CD8^+^ T cell responses. However, the durability of T cell responses was not strong, possibly due to the suppressive effects of regulatory T cells. Additionally, the study found that T cells had different specificity for various regions of the NY-ESO-1 protein at different time points, which may be due to the effect of regulatory T cells on different subpopulations of effector T cells, resulting in changes in T cell responses to specific peptides (33).

SCIB2 is an antibody DNA vaccine that encodes the NY-ESO-1 antigen and covers over 80% of HLA phenotypes by encoding 16 NY-ESO-1 epitopes (34). Compared to peptide vaccines, it generates an increased frequency and enhanced affinity of T cell responses, which exhibit potent cytotoxic activity against tumor cells expressing NY-ESO-1. In mice, SCIB2 demonstrated the ability to effectively suppress the growth of B16-NY-ESO-1-expressing tumor cells, leading to a long-term survival rate of 35%. When administered in conjunction with Treg depletion, CTLA-4 blockade, or PD-1 blockade, the long-term survival rates of mice exhibited substantial improvements, reaching 56%, 67%, and 100%, respectively (34).However, clinical results of SCIB2 have not been reported yet.

DNA vaccine is simple, stable and cost effective. However, one of the initial worries regarding the use of DNA vaccines was the possibility of integrating into the human genome. According to FDA guidelines, the rate of plasmid integration in DNA vaccines should be significantly lower than the rate of spontaneous mutation. Moreover, based on the existing clinical trial results, the NY-ESO-1 DNA vaccine cannot elicit satisfactory immune responses. Consequently, due to the non-genomic integration advantages and the remarkable achievements of COVID-19 vaccines, there is a growing interest among researchers in exploring the potential of NY-ESO-1 mRNA cancer vaccines as a promising approach.

### 2.4 mRNA vaccines

The COVID-19 pandemic has brought widespread attention to mRNA vaccine technology (35, 36). In fact, the rapid development of COVID-19 mRNA vaccines may be attributed to extensive prior research on mRNA vaccines as a potential therapeutic approach for cancer in both preclinical and clinical trials (36). mRNA vaccines offer several advantages in the vaccination process: firstly, mRNA vaccines are readily degradable and exhibit a favorable safety profile, and secondly, mRNA vaccines are a non-infectious, non-integrating platform technology, thereby eliminating the potential risks of infection or insertional mutations. Thirdly, mRNA vaccines have the potential to stimulate both humoral and cellular responses; finally, mRNA vaccines can be produced quickly at low cost (36). mRNA cancer vaccines can be custom-designed to target specific tumor antigens expressed by cancer cells, stimulating robust *in vivo* immune responses mediated by T cells or B cells against the tumors (37, 38). Since the inception and validation of the first mRNA cancer vaccine (39), a multitude of clinical studies have provided evidence for the safety and efficacy of mRNA vaccines in cancer therapy.

Up to now, there are three mRNA cancer vaccines targeting NY-ESO-1 that have entered clinical stages, named BNT111, CV9201 and CV9202. BNT111 is a nano-liposomal mRNA vaccine developed by BioNTech. It was granted orphan drug designation by the US FDA in September 2021. BNT111 encodes 4 TAAs: NY-ESO-1, MAGE-A3, tyrosinase, and TPTE. These 4 TAAs are expressed in over 90% of skin melanomas and have high immunogenicity. A Phase I clinical trial (NCT02410733) evaluating the use of BNT111 alone or in combination with PD-1 inhibitors in patients with unresectable melanoma demonstrated favorable safety profiles and initial signs of anti-tumor efficacy. Among 50 patients, over 39 patients (75%) were found to have immune responses to one or more TAAs, and BNT111 was able to induce CD4^+^ and CD8^+^ T cell responses. 17 patients received combination therapy with BNT111 and anti-PD-1, of whom 6 (35%) had a partial response and 2 (12%) had stable disease. 25 patients received BNT111 monotherapy, of whom 3 (12%) had a partial response and 7 (28%) had stable disease (40). Based on the success of NCT02410733 trial, the BNT111 vaccine received fast track qualification in November 2021. A randomized phase II clinical trial (NCT04526899) is currently evaluating FixVac BNT111, either alone or in combination with the PD-1 antibody cemiplimab, for the treatment of unresectable stage III and IV melanoma patients.

In another Phase I/IIa clinical trial (NCT00923312), 7 patients with locally advanced disease and 39 patients with metastatic NSCLC were treated with 5 doses of intradermal injection of CV9201, an active vaccine encoding 5 antigens including NY-ESO-1, MAGE-C1/2, survivin, trophoblast glycoprotein and 5T4 (41). The results showed that 63% of patients exhibited specific immune responses to one or more TAAs, and 60% of patients showed increased activation of IgD+CD38^high^ B cells. Disease stabilization (SD) was observed in 31% of patients, while the remaining patients exhibited disease progression (41). In a similar Phase I/IIa clinical trial (NCT03164772), CV9202 was administered in combination with local radiotherapy in patients with advanced NSCLC (42, 43). CV9202 is an RNA-based active vaccine encoding 6 TAAs including NY-ESO-1, MAGE-C1, MAGE-C2, 5T4, survivin, and MUC-1. The results showed that compared to baseline, 80% of patients experienced an increase in antigen-specific antibody levels, 40% of patients showed an increase in functional T cells, and 52% of patients exhibited evident involvement of multiple antigen-specific responses. One patient achieved PR with combined therapy of pembrolizumab, and 46.2% of patients achieved SD (42).

The development of NY-ESO-1 mRNA cancer vaccines has progressed rapidly, and future improvements may focus on achieving higher expression levels and prolonged expression time specifically within the target tissue.

### 2.5 protein and peptide vaccines

Protein- and peptide-based cancer vaccines are a form of immunotherapy that stimulate an immune response against TAAs or tumor-specific antigens (TSAs) by utilizing purified, recombinant, or synthetically engineered epitopes and proteins to trigger host immune responses (44, 45).

The NY-ESO-1 protein and peptide cancer vaccine trials have undergone significant advancements since their initial clinical trials over a decade ago. The discovery of proteins and the formulation of vaccines have been improved greatly, resulting in a range of synthetic peptides, recombinant proteins (both individual and complexed), and adjuvant formulations. Numerous clinical trials evaluating NY-ESO-1 recombinant protein vaccines have been conducted (**Table 1**). However, nearly none of these trials have advanced to phase III. The use of recombinant proteins can result in suboptimal or non-specific immune responses due to protein misfolding and inadequate epitope presentation (46–48). Thus, some researchers have redirected their attention towards NY-ESO-1 peptide vaccines (**Table 1**), Peptides derived from the NY-ESO-1 antigen bypass the requirement for protein antigen expression and processing, allowing for direct loading of epitopes onto MHC-I/MHC-II molecules. Currently, researchers have identified 21 unique epitopes that are restricted to at least 5 different HLA-class II alleles in the NY-ESO-1 antigen (3). Among these epitopes, NY-ESO-1_80–109_ and NY-ESO-1_157–165_ peptides demonstrated the highest immunogenicity, eliciting both CD4^+^ and CD8^+^ T cell responses (49). However, short peptides are poorly immunogenic and require adjuvants for enhanced and prolonged immune responses (50, 51). Various adjuvants have been extensively evaluated in conjunction with the NY-ESO-1 peptides and protein vaccines in clinical trials (48, 49, 52–66). These adjuvants include TLR agonists, such as OK-432, CpG, poly-ICLC, or MIS416, saponin-based adjuvant (ISCOMATRIX), incomplete Freund’s adjuvant (IFA) Montanide ISA-51. In a phase I clinical trial (NCT00616941) (55, 67), researchers investigated the impact of poly-ICLC and Montanide adjuvant on pre- and post-vaccine NY-ESO-1-specific CD4^+^ T cells. Vaccination with overlapping long peptides (OLP) from NY-ESO-1 alone did not induce CD4^+^ T cell responses. However, emulsifying OLP in Montanide was necessary for expanding high-avidity NY-ESO-1-specific CD4^+^ T cell precursors. Additionally, poly-ICLC significantly enhanced CD4^+^ Th1 responses while suppressing the generation of interleukin (IL)-4-producing Th2 and IL-9-producing Th9 cells (55). Another clinical trial was conducted to evaluate the safety and efficacy of CHP-NY-ESO-1 with MIS416 adjuvant in patients with refractory solid tumors expressing NY-ESO-1 (57). A total of 26 patients were enrolled in the study, and 7 patients (38%) continued receiving vaccinations during the maintenance phase. Throughout the trial, 6 patients (23%) experienced grade 3 drug-related adverse events, with one patient exhibiting anorexia and 5 patients experiencing hypertension. No grade 4-5 drug-related adverse events were reported. Among the patients, 8 (31%) demonstrated SD.

Additionally, NY-ESO-1 vaccination is being combined with other treatments, such as the mTOR inhibitor Sirolimus (NCT01522820), Decitabine (NCT01673217, NCT03358719), Azacitidine (NCT02750995), Cyclophosphamide (NCT00819806, NCT00518206 (68)), IDO1 inhibitor Epacadostat (NCT02166905) and PD-1 antibody Nivolumab (NCT05479045, NCT01176461 (69), NCT02737787, NCT01176474, NCT03358719) etc. And further investigation is needed to explore the potential of NY-ESO-1 protein and peptide vaccine in combination with other treatments to enhance the efficacy of the therapy.

The protein and peptide vaccines are simple, cheap to manufacture and stable when transported, which makes large-scale manufacture and transportation possible. Preclinical and clinical results have demonstrated the potency of NY-ESO-1 protein and peptide vaccines. However, the efficacy of individual peptide vaccines is often limited to specific HLA subtypes. Consequently, patients who do not express the commonly targeted HLA types may not be eligible for treatment with this vaccine. Besides, the immune response induced by synthetic peptide-based cancer vaccines may not accurately reflects or complements the natural immune response to endogenous antigen expression. Studies comparing the TCR features of naturally and vaccine-elicited NY-ESO-1 specific CTLs have indicated that these cells exhibited a highly conserved structure but distinct TCR features (70). These results indicate that the synthetic peptides used for vaccination may fail to accurately reflect the naturally processed antigen and antitumor immune response (3, 70). Since the vaccine peptides might not be the ones naturally processed. Thus, the actual presence on the tumor cells of the peptides used in vaccines should better be validated. Further research is needed to determine the optimal approach for developing effective NY-ESO-1 cancer vaccines that accurately reflect the natural immune response.

### 2.6 Therapeutic whole-tumor cell vaccines

The whole-tumor cell vaccine consists of genetically modified human tumor cell lines, which can be either autologous or allogeneic tumor cells. This vaccine approach exposes the immune system to a diverse range of tumor antigens, many of which are often unknown. To date, no therapeutic whole-tumor cell vaccine targeting NY-ESO-1 has advanced to the clinical stage. In a preclinical study, Xu et al. genetically engineered Renca cancer cells to express NY-ESO-1 and injected them into the renal cancer tumor (lacking NY-ESO-1) mice model (71). After treatment, the tumor size was significantly reduced in comparison to the control group. This reduction was attributed to the increased interaction between DCs and T cells with the NY-ESO-1 expressed Renca cancer cells. This suggests that NY-ESO-1 may be effective in training T cells to recognize and target the tumor-specific epitopes with high immunogenicity, even in tumors lacking NY-ESO-1, if coupled with the appropriate antigens.

### 2.7 Viral vaccines

Recombinant virus can act as vectors to express antigen gene, which represent a promising platform for vaccines. As the immune system has evolved to efficiently control viral infections, viral gene products may activate APCs, such as DCs, by triggering PRRs. At present, several viruses have been exploited as cancer vaccine platforms for encoding NY-ESO-1 antigens. NY-ESO-1 encoding recombinant fowlpox and recombinant vaccinia have been evaluated in clinical trials (72). Considering host-neutralizing immunity of subsequent vaccinations, the most viral vaccines, such as adenoviruses, vaccinia, MVA and other mammalian poxviruses, can only be given once. On the contrary, multiple injections of recombinant avipox (i.e., fowlpox) have been shown to induce non-neutralizing antibodies. Therefore, the recombinant fowlpox can be used for multiple booster vaccinations. In a clinical trial conducted under the Cancer Vaccine Collaborative (NCT00112957) (72), researchers used recombinant vaccinia-NY-ESO-1 (rV-NY-ESO-1) as prime and recombinant fowlpox-NY-ESO-1 (rF-NY-ESO-1) as booster in patients with various advanced solid tumors, and this diversified prime-boost regimen was certified to be safe and successfully induced both humoral and cellular immune responses specific to NY-ESO-1 in the majority of patients. Moreover, two parallel phase II clinical trials were conducted in 25 melanoma and 22 epithelial ovarian cancer (EOC) patients with advanced disease who were at high risk for recurrence/progression to test the clinical efficacy of rV-NY-SO-1 and rF-NY-ESO-1 prime-boost regimen (73). The results showed that a significant proportion of melanoma and EOC patients exhibited induction of NY-ESO-1-specific antibody, as well as CD4^+^ and CD8^+^ T cells. In the melanoma patients, CR rate was 9.5% (2/21), PR rate was 4.8% (1/21), mixed response rate (MR) was 4.8% (1/21), and SD rate was 52.4% (11/21), resulting in a clinical benefit rate (CBR) of 71.5%. In the melanoma patients, the median PFS and the median OS was 9 months and 48 months, respectively. In the 22 EOC patients, the median PFS and the median OS was 21 months and 48 months, respectively (73).

vCP2292 [ALVAC (2)-NY-ESO-1(M)-TRICOM] is a recombinant ALVAC (2) poxviruse containing transgenes NY-ESO-1 and co-stimulatory molecules (TRICOM: B7-1, ICAM-1, and LFA-3). TRICOM genes have been shown to have the ability to induce higher numbers of antigen-specific T cells, enhance the avidity of these T cells, and improve tumor activity (74). In preclinical trials, it was observed that vCP2292 could elicit NY-ESO-1 specific T cell responses in mice. Unexpectedly, vectors lacking TRICOM generated higher NY-ESO-1-specific responses than those containing TRICOM. This could be due to difference between human TRICOM with its murine counterpart, and the human TRICOM inserted in the vectors was recognized as foreign antigen in the mice, the immune response against the foreign human TRICOM was further competed with the anti-TAA responses, leading to a reduction in the responses elicited by vectors containing TRICOM. However, this effect is not considered worrisome in a human context, as no immune responses against TRICOM were observed in clinical trials involving other vectors carrying human TRICOM (74).

As cancer advances, malignant cells have the ability to evade detection by the immune system. Boehmer et al (75) found the existence of immunoediting and immune escape in a melanoma patient, whose primary tumor displayed expression of NY-ESO-1, MAGE-C1, and Melan-A. The patient was immunized with a recombinant NY-ESO-1 fowlpox vaccine followed by immunization with NY-ESO-1 protein + CpG. Spontaneous humoral and cellular responses against NY-ESO-1 were observed, which were further enhanced by subsequent immunizations. However, despite induction of the immune responses, in the following years, all developing lesions were found to be negative for NY-ESO-1, while being positive for MAGE-C1, Melan-A, and MHC-I (75). Thus, a multivalent NY-ESO-1 vaccine (including two or more cancer antigens) might be more effective at preventing the outgrowth of NY-ESO-1-negative tumors.

The virus-based NY-ESO-1 vaccines demonstrate natural immunogenicity, and the oncolytic virus vaccines exhibit the capacity to directly eradicate tumor cells. However, the emergence of neutralizing antibodies in patients and safety concerns may impose limitations on its clinical application.

## 3 Conclusion and perspectives

NY-ESO-1 is a potential prognostic factor and a promising target for immunotherapy. With an enhanced comprehension of antitumor immune mechanisms, various approaches, including cancer vaccines, adoptive T cell therapy (ACT) and combination therapies targeting NY-ESO-1 against solid cancers have made significant progress in recent years. Cancer vaccines targeting NY-ESO-1 have come a long way, different types have shown promising results in preclinical trials, leading to the initiation of new clinical trials for the treatment of solid cancers. Currently, there are more than 70 clinical trials registered using NY-ESO-1-based cancer vaccines, and they have gained significant attention in clinical prevention and treatment. While there have not yet been any successful clinical applications of NY-ESO-1-based cancer vaccines, it is crucial to note that they hold significant potential for antitumor applications following additional refinement and assessment in clinical trials. Safety and effectiveness are crucial factors that must be considered in all preclinical and clinical studies. To ensure the successful development of cancer vaccines targeting NY-ESO-1 in clinical trials, several critical questions must be addressed. Firstly, it is essential to activate and balance both the innate and adaptive immune responses through various mechanisms and pathways. Secondly, the enhanced immune monitoring function after vaccination is essential to accurately assess the vaccine’s efficacy. Finally, the issue of tumor immune evasion must be addressed, as tumors can downregulate antigen presentation or induce immune suppression, which can limit the effectiveness of tumor vaccines.

As the self-antigens are expressed in the thymus, the T cell repertoire of CD8^+^ or/and CD4^+^ T cell against self-antigen will be blunted by central tolerance (76), the immunogenicity of cancer vaccines targeting NY-ESO-1 in patients with advanced cancer may be affected by the immuno-editing and tumor evasion. To address this issue, future research efforts should focus on optimizing the immune microenvironment to enhance the activation of immune cells and promote tumor recognition and rejection. Additionally, the protective immune effect of cancer vaccines targeting NY-ESO-1 can be influenced by the immunization route. Therefore, further investigation into the optimization of immune processes, improvement of vaccine design and the potential advantages of cancer vaccines targeting NY-ESO-1 is warranted.

As a monotherapy, cancer vaccines have not yet been demonstrated to show outstanding clinical results in malignant tumor treatment. Recently, there has been a growing interest in the integration of cancer vaccines with ACTs to combat tumor evasion of immune surveillance and resistance (77–79). Ma et al (77, 78) identified that cancer vaccination induces metabolic changes in CAR-T cells, leading to an increase in the production of interferon gamma (IFN-γ), which may potentially contribute to overcoming the immunosuppressive tumor microenvironment. Furthermore, the enhanced efficacy of CAR-T cells induced by tumor vaccination may be associated with DCs, including factors such as DCs recruitment, uptake of tumor antigens, and activation (77, 78). In another study, researchers discovered that the combination of tumor vaccines with TCR-T cell therapy significantly enhanced the anti-tumor response of TCR-T cells and induced epitope spreading among the endogenous T cell population, leading to durable eradication of established solid tumors in syngeneic tumor models (79). The improved anti-tumor efficacy was associated with pro-inflammatory transcriptional reprogramming in lymph nodes and enhanced maturation of antigen presenting cells. This led to the expansion and functional enhancement of TCR-T cells in both lymph nodes and the solid tumor tissue, without the need for lymphodepletion (79). It is important to note that the aforementioned conclusions are based on experimental results obtained from mouse models, further research and validation are required to determine the feasibility and effectiveness of these findings in clinical applications. Additionally, it is crucial to delve deeper into the mechanisms underlying the interaction between vaccines and ACTs in order to optimize immune therapeutic strategies.

## Author contributions

MW: Writing – review & editing. HZ: Writing – original draft. YM: Writing – review & editing. FL: Writing – review & editing. BL: Writing – review & editing. DQ: Writing – review & editing. PR: Writing – review & editing.
